# Biology of neurofibrosis with focus on multiple sclerosis

**DOI:** 10.3389/fimmu.2024.1370107

**Published:** 2024-03-26

**Authors:** Brian M. Lozinski, Samira Ghorbani, V. Wee Yong

**Affiliations:** Hotchkiss Brain Institute and the Department of Clinical Neuroscience, University of Calgary, Calgary, AB, Canada

**Keywords:** neurofibrosis, fibrosis, CNS, scarring, ECM, MS

## Abstract

Tissue damage elicits a wound healing response of inflammation and remodeling aimed at restoring homeostasis. Dysregulation of wound healing leads to accumulation of effector cells and extracellular matrix (ECM) components, collectively termed fibrosis, which impairs organ functions. Fibrosis of the central nervous system, neurofibrosis, is a major contributor to the lack of neural regeneration and it involves fibroblasts, microglia/macrophages and astrocytes, and their deposited ECM. Neurofibrosis occurs commonly across neurological conditions. This review describes processes of wound healing and fibrosis in tissues in general, and in multiple sclerosis in particular, and considers approaches to ameliorate neurofibrosis to enhance neural recovery.

## 1 Introduction: impaired wound healing contributes to tissue fibrosis

A proper wound healing response is vital for tissue regeneration and involves discrete stages of inflammation, tissue remodeling, and their resolution (1). The inflammatory response is critical for the removal of pathogens and debris from the injury site. It also recruits and activates tissue specific effector cells required for remodeling. Immune and effector cells deposit a variety of extracellular matrix (ECM) molecules to reconstitute matrix lost to injury (1). Successful wound healing recapitulates the tissue environment and restores function (2). Dysregulation of wound healing following repeated, chronic, or pronounced single injury leads to fibrotic scarring due to excessive build-up of cells and ECM (1, 3).

**Figure 1:**
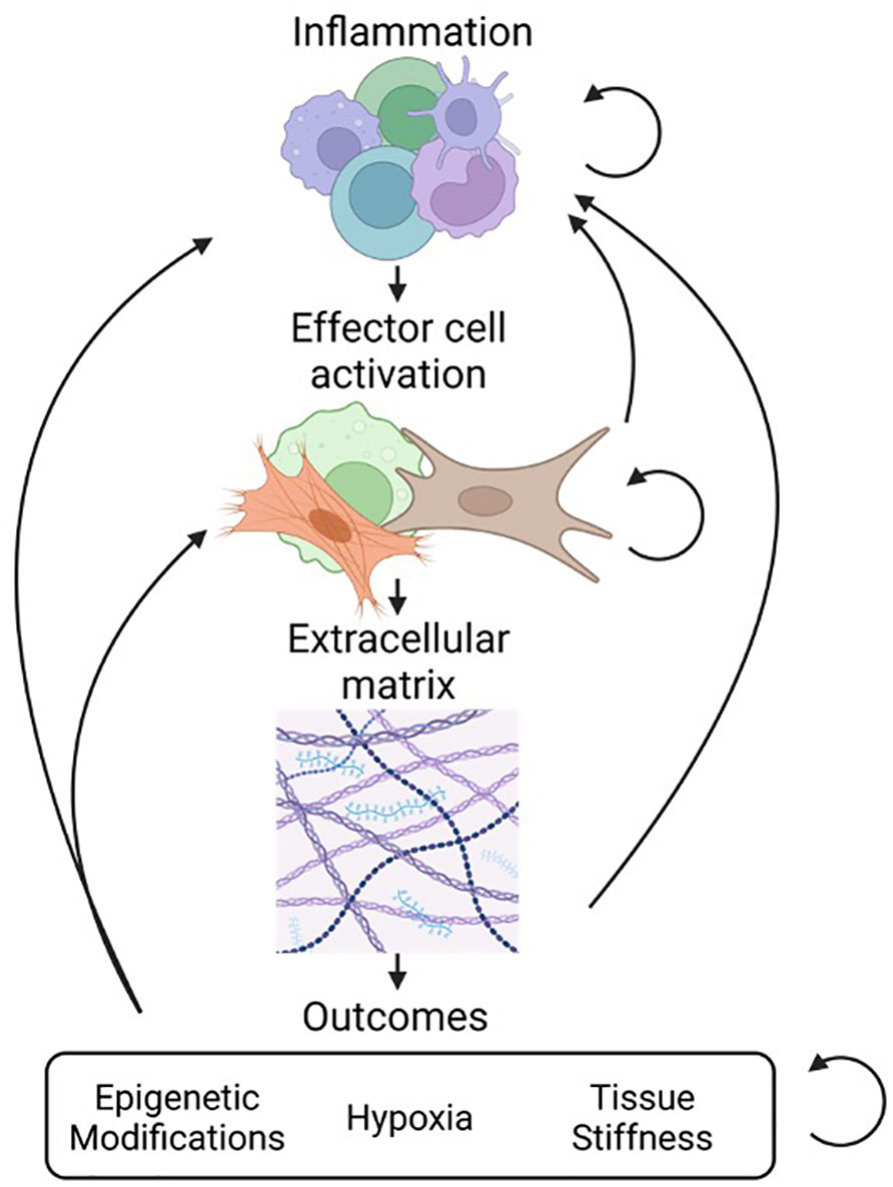
Overview of fibrosis and interconnectivity of stages. Fibrosis progresses through several stages. The first stage of inflammation involves accumulation of a heterogeneous population of cells such as macrophages, dendritic cells, and lymphocytes (top). This is followed by the activation of effector cells such as fibroblasts and myofibroblasts that drive the process of tissue remodeling (middle). The result of effector cell activation is the production and deposition of ECM components including collagen, fibronectin, laminins, and proteoglycans (bottom). As a result of fibrosis there can be secondary injury that results from epigenetic modification of cells, tissue hypoxia, and increased tissue stiffness.

Fibrosis is characterized by increased tissue stiffness and disrupted tissue architecture (**Figure 1**) (3). It is identified by the accumulation of ECM components, altered protease expression, increased levels of pro-fibrotic signaling molecules, and the presence of pro-fibrotic cells such as fibroblasts and their differentiated forms, myofibroblasts (1). Even in conditions not classically associated with fibrosis, disease outcomes such as hypoxia and epigenetic reprogramming of cells are described with fibrosis-linked responses (4) such as progressive scarification and exacerbated tissue injury (5).

In the central nervous system (CNS) fibroblasts occupy border regions such as the meninges and perivascular space (6). Following injury they become elevated in the parenchyma where they, along with astrocytes, microglia, infiltrating immune cells and potentially pericytes contribute to tissue reorganization and ECM accumulation (7, 8). This neurofibrosis response occurs commonly in CNS pathologies such as spinal cord injury (SCI), stroke and multiple sclerosis (MS) (9). Here we discuss general wound healing and fibrosis related responses so as to instruct concepts of neurofibrosis, and we then consider fibrosis in the CNS with a focus on MS. Finally, we evaluate the therapeutic potential of targeting neurofibrosis to improve outcomes from CNS injuries.

## 2 Wound healing and fibrosis related responses

**Figure 2:**
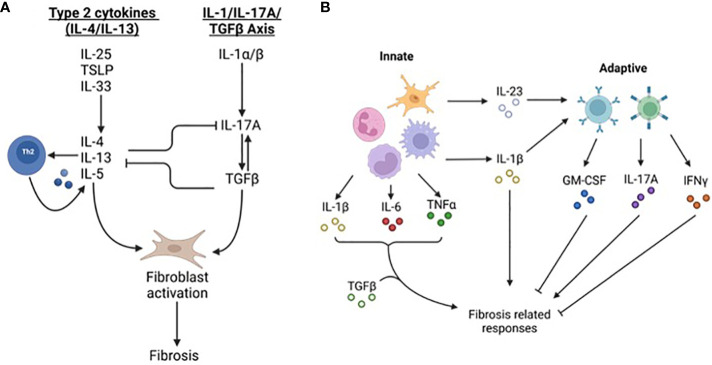
Fibrosis-related signaling pathways. **(A)** Fibrosis canonically occurs through ‘type 2 cytokines’ including IL-4 and IL-13 and the IL-1/IL-17/TGF-β signaling axis. Danger-associated molecular patterns including thymic stromal lymphopoietin (TSLP), IL-25 and IL-33 stimulate the production of IL-4, IL-5, and IL-13. These cytokines cause T_h_2 cells to produce more cytokines including IL-4/IL-13, macrophages to produce TGF-β, and stromal cells, including fibroblasts, to elevate and deposit ECM components. The second pathway stimulating fibrosis is the IL-1/IL-17/TGF-β axis. IL-1, along with other inflammatory cytokines such as IL-6 and TNF-α, stimulate IL-17A expression in neutrophils and T cells. IL-17A increases expression of TGF-β and fibroblast expression of TGFβRIII leading to greater fibrosis. **(B)** Fibrosis is regulated by innate and adaptive immunity. Early phases of fibrosis are influenced more by innate immune cells including macrophages, neutrophils, monocytes, and microglia (in the CNS). Cytokines such as IL-1β, IL-6, and IL23 affect both fibrosis and immune responses. During later stages of fibrosis lymphocyte derived IL-17A, IFN-γ, and GM-CSF promote and impede fibrosis-related responses. IFN-γ may impede fibrosis by limiting T_h_2 differentiation and type 2 cytokine signaling but can contribute to fibrosis by stimulating immune infiltration leading to greater tissue injury. GM-CSF is anti-fibrotic and dampens pro-fibrotic cytokine production and alternatively activated macrophage polarization.

Fibrosis occurs in many organs and has shared and unique tissue specific characteristics (4). The initiating injury varies based on anatomical location and trigger (5). For instance, liver and kidney fibrosis can occur due to viral hepatitis and diabetes, respectively. Resident and infiltrating immune cells contribute to fibrosis during initial innate and later adaptive responses (3) (**Figure 2**). Two primary signaling axes, interleukin (IL)-4/IL-13 and IL-1/IL-17A/transforming growth factor-β (TGFβ), are core pro-fibrosis pathways (5), although IL-17 may also be anti-fibrotic depending on the triggering insult and the organ affected (10). Macrophages, the most abundant immune cell type in fibrosis, are recruited by damage associated molecular patterns (DAMPs), pathogen associated molecular patterns (PAMPs), and chemokines (11). Early arriving macrophages possess an inflammatory phenotype and secrete pro-inflammatory tumor necrosis factor-α (TNFα) and IL-1β which fuel inflammation (11). Later stage macrophages assume a tissue remodeling regulatory phenotype characterized by the expression of TGFβ, IL-10, and mannose receptor 1(MRC1/CD206) (11). Inflammatory macrophages contribute to fibrosis by exacerbating the injury, while regulatory macrophages stimulate effector cells to deposit ECM and express other tissue remodeling genes (5). It is not clear what dictates IL-4/IL-13 or IL-1 mediated fibrosis although the chronicity of the injury may be a determinant. Single injections of bleomycin produce fibrosis in an IL-1/IL-17/TGFβ dependent manner; conversely, repeated injections of bleomycin over a longer period elicits IL-4R signaling (5). This has repercussions for signaling to effector cells and the transition to the tissue remodeling stage of repair.

Effector cells responsible for tissue remodeling originate from resident and/or recruited fibroblasts, epithelial cells, endothelial cells, and other tissue resident cells and immune cells (4). Effector cells are recruited by chemokines and growth factors elaborated at sites of injury by the initial-arriving immune cells where they then proliferate and upregulate ECM, proteases, and growth factors (12). Positive feedback loops between effector and immune cells and the fibrotic environment promote further fibrosis by elevation of pro-fibrotic genes (13, 14). For example, fibroblasts upregulate ECM genes when cultured on ECM derived from idiopathic pulmonary fibrosis tissue (14). This can lead to greater scarring of the tissue and disruption of function.

Organ failure and increased morbidity are outcomes of fibrosis-related disorders such as cardiac fibrosis following myocardial infarction (5). Prominently, fibrosis-related disorders are responsible for 45% of fatalities in the United States of America (5). This highlights the severity of fibrosis for tissue function and recovery which is made more significant in a tissue environment that is not prone to regeneration, such as the CNS.

## 3 Neurofibrosis

Functional regeneration of the CNS does not occur effectively in adult humans (15) due to factors including lack of available stem cells, age-related changes in neural cells and the tissue environment, and the formation of inhibitory scar tissue after injury (16, 17). Scarring of the CNS results from the accumulation of astrocytes, microglia and macrophages, and fibroblasts and potentially pericytes. The formation of neurofibrosis contributes to impairment of axonal regrowth due to elevated deposition of collagens and chondroitin sulfate proteoglycans (CSPGs), amongst others, into the lesion (7, 8).

**Figure 3:**
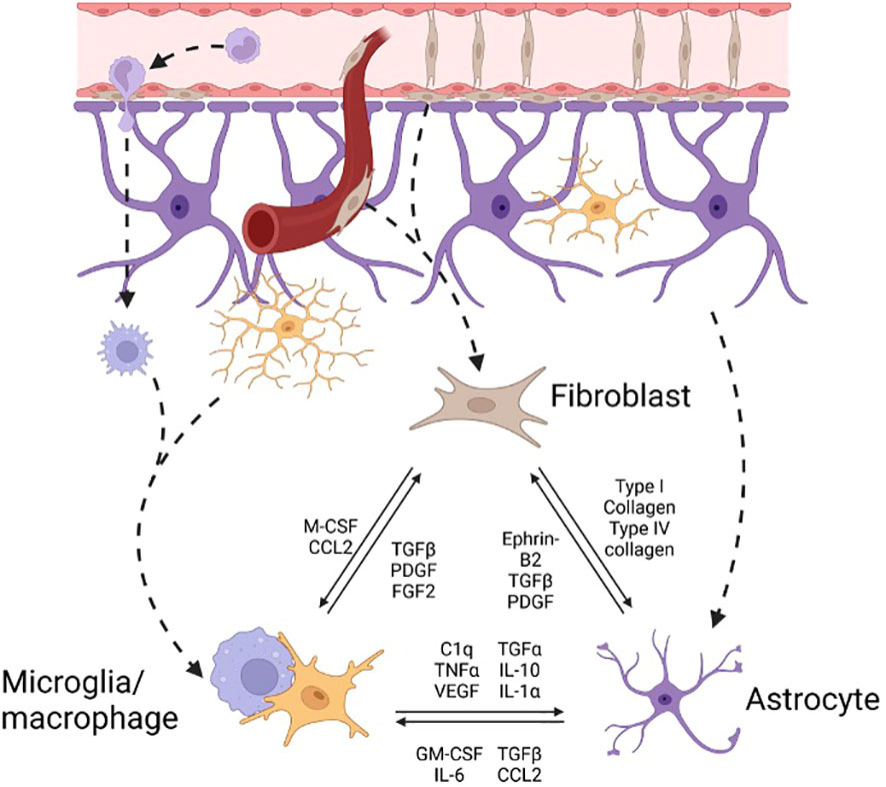
Effector cell interactions in neurofibrosis The pool of effector cells in CNS fibrosis is drawn from circulating monocyte derived macrophages, border derived meningeal and perivascular fibroblasts, and resident microglia and astrocytes. There is significant interconnectedness between cell types. cell-cell, cell-ECM-cell, and soluble factor signals between effector cells leading to reduced or greater stimulation of fibrosis-related pathways such as TGF-β signaling in fibroblasts or collagen-1 activation of astrocytes.

In SCI astrocytes become reactive and form a protective boundary to prevent spread of the injury while microglia/macrophages and fibroblast-like cells occupy the center (**Figure 3**). In MS lesions microglia/macrophages and astrocytes are more intermingled while fibroblast-like cells are expanded in the perivascular compartments (18). Conflicting descriptions of the beneficial and harmful roles of reactive astrocytes exist and have been elaborated on elsewhere (17). Though reactive astrocytes restrict the spread of injury and attempt to maintain homeostatic functions (17), they have also been described to contribute to chronic neuroinflammation and they upregulate many ECM components detrimental for regeneration (19). Conversely, depletion of reactive astrocytes impairs the ability of axons to regrow after SCI (20). Instructively, studies such as these suggest that certain levels of regeneration of the CNS are possible under permissive conditions.

CNS-associated fibroblasts identified by PDGFRβ and Col1a1 driven reporter in mice and antibody labeling are increasingly implicated in neurofibrosis (8, 21). Their complete depletion in SCI results in an open wound defect (8) while their partial depletion leads to greater axon regeneration (22, 23). The identification and ontogeny of fibroblasts in neurofibrosis remain to be better resolved, and the use of different reporter mouse lines is partly responsible for the controversy. Due to the use of PDGFRβ based reporter mice and PDGFRβ as a common marker across several cell types, it is likely that fibroblasts, pericytes, and smooth muscle cells have been conflated with each other (24). Transcriptional profiling of murine mural cells has shown that all these three cell types express common markers including PDGFRβ and NG2 (5, 24). While these studies identified homeostatic gene expression patterns that differentiate pericytes (*Kcnj8*), fibroblasts (*Col1a1*), and smooth muscle cells (*Acta2*), it is unclear how expression of these are affected by injury or inflammation. Further adding to the controversy, type A pericytes identified by a GLAST driven reporter line have been described in SCI (8, 25) but GLAST also labels populations of astrocytes. As well, Dorrier et al. showed using NG2 and SMA inducible reporters that cells basally positive for these genes do not significantly contribute to fibrotic cells in neuroinflammatory lesions (21, 22). However, this does not preclude subpopulations of pericytes or other cell types from contributing to neurofibrosis. Additionally, other lines of evidence suggest that pericytes and smooth muscle cells do not contribute to the fibroblast populations in neurofibrosis although the data is inconclusive (8, 21). Based on the uncertainty of the ontogeny or type of fibroblasts in CNS injuries, we shall refer to them as fibroblast-like cells where indicated.

The molecular impediments to CNS regeneration within fibrotic lesions include inhibitory molecules, reduced levels of growth signals and products of particular inflammatory cells (8, 17). Microglia are CNS resident macrophages and are critical for maintaining tissue homeostasis. Monocyte derived macrophages become elevated following injury and, together with microglia, participate in cellular and lipid debris removal. As well, they upregulate inflammatory and tissue remodeling genes during early and late stages of injury. The ability of microglia/macrophages to metabolize debris becomes impaired with age leading to formation of foamy cells that contribute to chronic inflammation and impaired tissue remodeling (16). In neonatal mice infiltration by peripheral macrophages is resolved rapidly compared to adult mice. Depleting microglia using PLX3397 or CX3CR1-CSF1R^fl/fl^ reversed spontaneous neonatal axonal regeneration implying that it is microglia that act to promote recovery following optic nerve crush injury (26). Additionally, there is a clear interconnectedness of the cells within CNS lesions as highlighted by altered levels of astrocytes, macrophages, and fibroblasts following depletion of each individually (22, 25, 27). Indeed, both astrocytes and fibroblasts interact reciprocally with immune cells informing each cell’s phenotype (**Figure 3**) (21, 28). This emphasizes the importance of the cellular components of CNS injury to the success of regeneration.

A theme that has been topical in peripheral fibrosis is that of fibroblast senescence and resistance to apoptosis (29, 30). Thus far, a systematic analysis of age-related neurofibrosis has not been thoroughly conducted. Studies focusing on the effects of age on traumatic brain injury described increased collagen levels in the injured aged meninges (31). Whether this is consistent in humans is not clear though proteomic analysis of CSF in people found increased levels of collagen with aging (32). Further determinations of changes with aging would include fibroblast density and susceptibility to apoptosis in lesions, accumulation of neural ECM in neurological disorders, and tissue rigidity in healthy aging or disease CNS.

While tissue stiffness generally increases in non-CNS fibrosis-related disorders, many forms of CNS injury are reported to result in reduced stiffness (33). One explanation for this phenomenon may be that most animal models of MS are studied during acute stages of injury and tissue stiffness increases during chronic stages of injury. Indeed, CNS lesions become increasingly stiff with chronicity (34). Thus, neurofibrosis is akin to fibrosis present in other organs and presents further questions about the role of fibrosis-related responses in CNS pathologies including MS.

## 4 MS and its regenerative processes

MS is an inflammatory disease characterized by demyelination and neuroaxonal degeneration in the brain, spinal cord, and optic nerve (35). Demyelination occurs in the context of inflammation involving CD8+ T lymphocytes, CD4+ T lymphocytes, B cells, plasma cells, macrophages, microglia and reactive astrocytes (35–37). Lesions are categorized as active, chronic active, inactive, and remyelinating based on immune and remyelinating phenotypes (16). Found in the brain and spinal cord white and gray matter, the location of lesions contributes to symptom presentation and onset (35).

Axonal regeneration can occur under ideal conditions, but it is not common and has only been minimally examined in MS. Conversely, remyelination occurs spontaneously to varying degrees in MS, in all types of lesions except inactive ones, and has been extensively examined (16, 38). During remyelination, oligodendrocyte progenitor cells (OPCs) migrate to the site of injury, proliferate, and differentiate into mature oligodendrocytes capable of producing new compact myelin (16). Remyelination promotes axonal neuroprotection and functional recovery making it an important and regularly occurring regenerative process (16, 38).

Fibrosis affects remyelination in MS (7). OPC migration, proliferation, and maturation are positively and negatively regulated by particular ECM components deposited in lesions (7). Specific effects of different ECM components have been reviewed in detail previously (39). Examples include beneficial effects of some isoforms of laminins on OPC proliferation while ECM molecules such as CSPGs and fibronectin impair OPC activity (7, 40). Inhibition of CSPG synthesis following injury increased oligodendrocyte numbers and remyelination (41). Furthermore, depletion of astrocytes and fibroblasts in models of MS leads to greater density of oligodendrocytes (21, 22). Thus, several elements of fibrosis impair regenerative processes in MS, to which we now turn our attention.

## 5 Biology of neurofibrosis in MS

Many processes dysregulated in fibrosis are present in MS lesions including chronic inflammation, effector cell recruitment, and excessive ECM deposition (5, 16). As well, outcomes of fibrosis such as tissue stiffness, hypoxia, cellular senescence, and impaired repair processes impact MS pathology (42, 43). Here we describe neurofibrosis in MS focusing on the inflammation, and remodeling and progression stages.

### 5.1 Inflammation

Lesion-associated cells in MS include microglia and astrocytes, infiltrated leukocytes, and CNS barrier-associated cells such as meningeal and perivascular macrophages and fibroblast-like cells (6, 35–37). CD8+ and, to a lesser extent, CD4+ T cells are present in MS lesions while B cells are within the perivascular and meningeal borders (37). Lymphocytes are contributors to MS pathology and have been detailed elsewhere (36, 37, 44).

The inflammatory milieu within MS lesions is diverse and shares many inflammatory characteristics seen in fibrosis. Pro-inflammatory cytokines including GM-CSF, IL-17, and IFNγ are elevated in MS and contribute to pathological processes (44). As well, TGFβ, the master regulator of fibrosis and important regulatory cytokine, is highly expressed in MS lesions (45). Both IL-4/IL-13 and IL-1 mediated fibrosis pathways converge through TGFβ. As mentioned previously, IL-4/IL-13 seem to be associated with persistent injury such as that seen in MS although the prominent pro-inflammatory nature of MS leads IL-4 and IL-13 to be often minimized in its pathophysiology. IL-1 on the other hand is highly expressed in MS lesions, as are T_h_17 cells and IL-17. Thus, the IL-1/IL-17/TGFβ pathway constituents are more prevalent in MS (46, 47). The increased IL-17 levels are important in sustaining inflammation by recruitment of myeloid cells (48), potentially producing a positive feedback loop for neurofibrosis. As well, IL-17 upregulates and stabilizes TGFβR (R: receptor) expression on fibroblasts (8, 17). This allows for TGFβ signaling through SMAD2/3 to promote activation of astrocytes and differentiation of fibroblasts into myofibroblasts positive for αSMA, collagen, and fibronectin (4, 20).

Microglia/macrophages are the most prevalent immune cells in MS lesions. Fibrosis-associated microglia/macrophages expressing arginase-1, MRC1/CD206, and MerTK (49, 50) have been described in MS lesions (35, 51). Important for the onset of fibrosis, microglia and macrophages become polarized to arginase-producing cells by their local environment after initially expressing nitric oxide synthase (51, 52). Arginase 1 converts arginine into precursors of proline essential for collagen production (4, 50). As well, macrophages upregulate proteases in response to T_h_1 derived cytokines such as IFNγ (53). IFNγ may also impair collagen synthesis by fibroblasts and reduce pro-fibrotic IL-17 expression (1, 44). However, in the presence of IL-6, IFNγ can help promote fibrosis in a STAT1 dependent manner (54). Thus, it is not clear whether IFNγ will behave as a pro- or anti-fibrotic cytokine in MS though IFNγ and IL-6 are commonly described in MS and may favor an avenue to progression of fibrosis (55).

**Figure 4:**
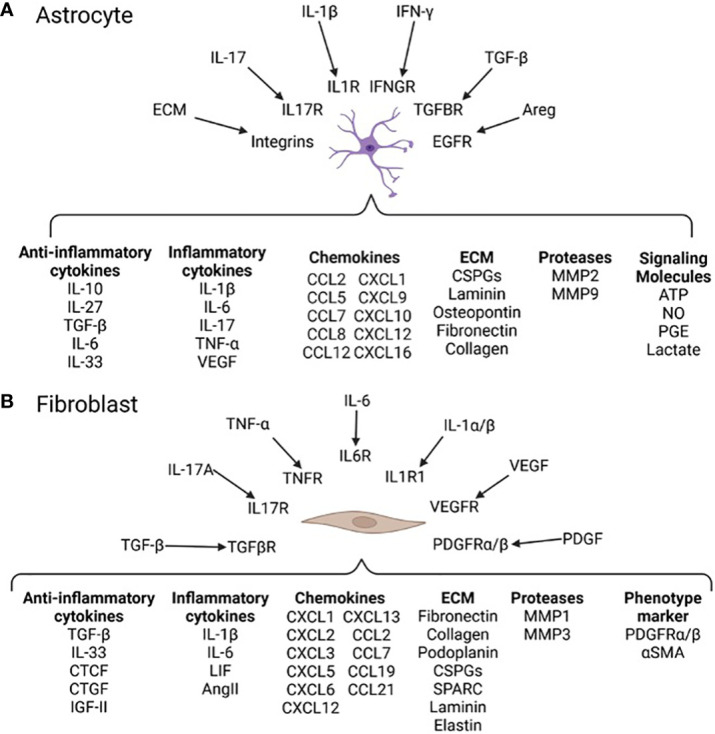
Neurofibrosis related signals and responses in astrocytes and fibroblasts **(A)** Soluble factors and ECM components such as IL-1β and collagen can be recognized by astrocytes through cell surface receptors (17). Activation of secondary signaling cascades polarize astrocyte phenotypes affecting production of pro- and anti-fibrotic proteins. For instance, autocrine LacCer signaling induces astrocyte expression of NFkB and downstream genes such as CCL2 and GM-CSF leading to greater inflammation (19). **(B)** Fibroblasts reside in regions of the CNS in close proximity to immune infiltrates allowing them to sample inflammatory factors such as IL-17A and TNF-α (8). Fibroblasts express a range of genes related to immune modulation, contractility, and tissue remodeling (8, 56). Activated fibroblasts transition to a tissue remodeling myofibroblast phenotype expressing contractility proteins (e.g. αSMA) and producing ECM such as collagen and fibronectin (8). The specific outcomes of this in MS and other neurological diseases is not well understood.

Astrocytes express pro- and anti-inflammatory cytokines in a context dependent manner and contribute to neurofibrosis. In chronic experimental autoimmune encephalomyelitis (EAE), a model of MS, astrocytes promote inflammation, immune cell recruitment, and further astrocyte reactivity by production of GM-CSF, CCL2 and lactosylceramide (19). The promotion of chronic inflammation during late stage EAE by reactive astrocytes contributes to worse disease scores and is reversed following depletion of reactive astrocytes (19). As well, IL-17-NFκB signaling in astrocytes results in expression of pro-inflammatory cytokines, chemokines, and proteases (**Figure 4A**). Astrocytes are also a source of IL-6 and vascular endothelial growth factor (VEGF) which stimulate macrophages and fibroblasts towards pro-fibrotic phenotypes (57). It is reasonable to surmise that while astrocytes may limit fibrosis early in EAE (8), they contribute to injurious neuroinflammation that promotes fibrosis during chronic stages of EAE and likely MS.

### 5.2 Tissue remodeling and progression of fibrosis in MS

Tissue remodeling is driven by effector cell recruitment and activation (4). Effector cells in MS lesions include microglia/macrophages, astrocytes, perhaps pericytes, and perivascular and meningeal fibroblasts (7, 21). Indeed, these cells are associated with ECM deposition in MS and animal models of MS (7, 8). Proteomic and histological analysis of active, chronic active, and inactive MS lesions highlight the involvement of fibrosis and ECM components (42, 55). The latter include fibronectin, proteoglycans and thrombospondin particularly in chronic active lesions (55). Importantly, molecular network analyses implies integrin signaling is involved within both chronic active and inactive lesions indicating a role for integrin-interacting ECM in resolving or following the resolution of inflammation (55). As noted above, key regulators of ECM expression such as TNFα, IL-17, TGFβ, IFNγ, IL-1, and platelet derived growth factor (PDGF) are elevated in MS lesions (55, 58).

Microglia/macrophages are found in MS lesions in close proximity of accumulated ECM (7). As well, microglia/macrophages undergo transition from inflammatory to remodeling phenotypes in MS and models of MS that coincides with a transition from inflammation to tissue remodeling (16). Depleting microglia/macrophages during tissue remodeling impairs remyelination in part due to the loss of Activin A mediated OPC maturation (51). During early inflammation, depleting microglia/macrophages causes myelin debris accumulation and reduced OPC proliferation (28, 51). Interestingly, type 1 interferons associated with viral infection and aging cause microglia/macrophages to express fibrosis related genes (37). A direct connection between tissue stiffness and disease progression in MS in not known, but increasing tissue stiffness is associated with age and lesion chronicity and may suggest a role of altered ECM (43, 59).

Astrocytes tile the CNS making them ideal effector cells (17). Following injury they upregulate a plethora of tissue remodeling and ECM components including hyaluronan, fibulin-2, CSPGs, and laminins (18, 60). Expression of these molecules relies in part on TGFβ and EGF signaling (7, 61). During chronic EAE, astrocytes upregulate tissue remodeling associated genes *Arg1*, *Spp1* (which encodes the matrix associated protein osteopontin), and *Vegf* (19) that may lead to enhanced fibrosis.

In MS, fibroblasts are associated with the perivascular space and increased expression of basement membrane and mesenchymal cell markers (21, 62). During EAE they are found in regions high in microglia/macrophages and express tissue remodeling related genes for ECM, proteases, and cytoskeleton proteins (21). Lesion-associated fibroblasts express many molecules (**Figure 4B**) including high levels of collagens and fibronectin (21), which impair the function of OPCs (7). As well, these, and other, ECM components can feedback into fibrosis-related pathways by acting as agonists for TLR signaling of microglia/macrophages, exacerbating inflammation and subsequent fibrosis (7).

Despite the prominence of fibroblasts in non-CNS fibrosis disorders, their description in MS lesions has thus far been limited. Reports identify fibroblasts in brains of people with MS in the meninges and perivascular space, and sparsely in the parenchyma (9, 21). This may be due to poor availability of fibroblast markers, altered marker expression, or they may be transient populations within the lesion environment. Further work is clearly needed to characterize and understand the contribution of fibroblasts to MS lesions.

## 6 Emerging/potential therapeutics

**Figure 5:**
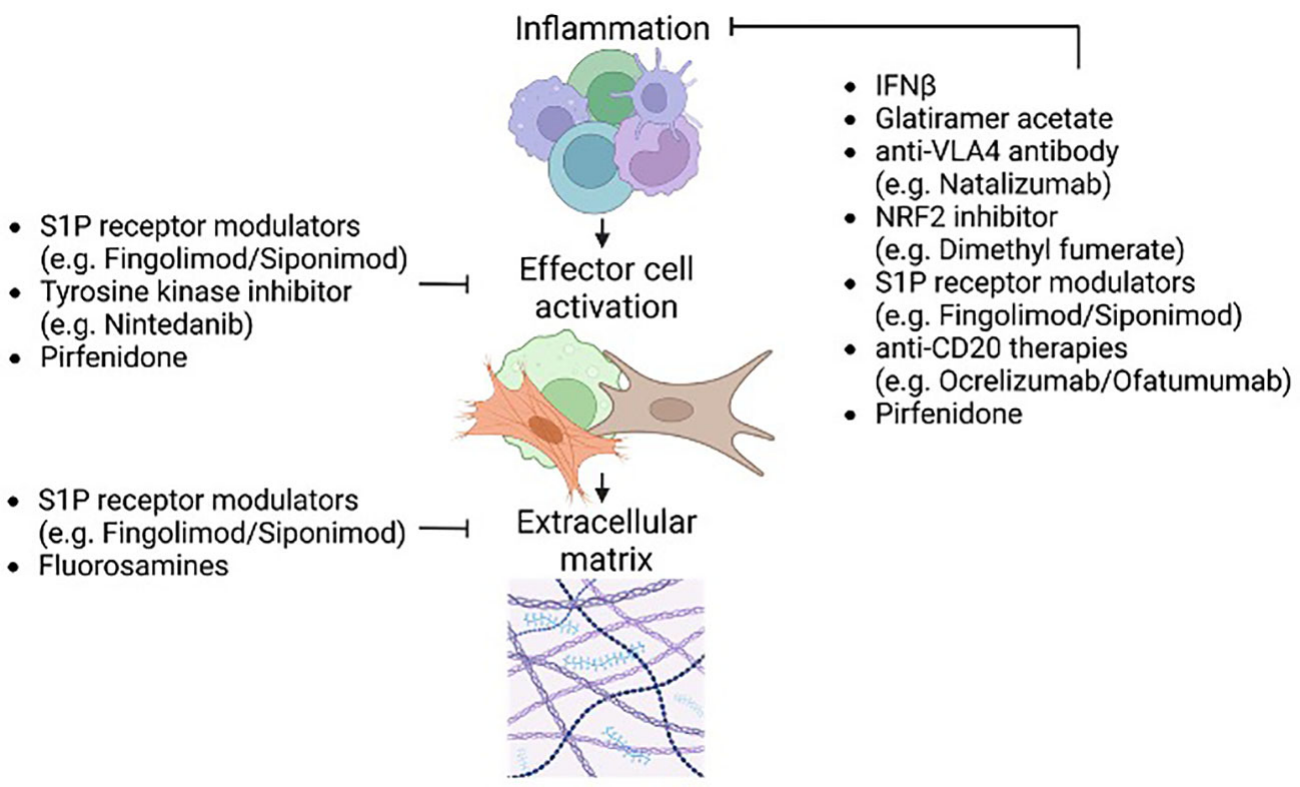
Effects of existing MS disease-modifying therapies (DMTs) on fibrosis pathways. Existing MS related DMTs may affect all stages of fibrosis. Due to the neuroinflammatory nature of MS the inflammatory component of fibrosis is most affected by DMTs. Impeding inflammation serves to limit inflammatory tissue injury and the potential for unchecked remodeling during later lesion stages. Furthermore, impeding the initial inflammatory stages of fibrosis has downstream effects on the later fibrosis-related processes such as ECM deposition and remodeling. Some current and potential DMTs including S1P receptor modulators and pirfenidone target activation of effector cell populations. This includes but is not limited to affecting TGFβ signaling important for effector cell activation and the development of fibrosis. Affecting effector cell activation directly affects ECM deposition and secondary injury caused by elevated stiffness and hypoxia. Preliminary studies on therapeutics that target the final stages of fibrosis such as ECM synthesis (e.g. fluorosamines) highlight the benefits of altering later stages of neurofibrosis.

No therapies currently are used to directly affect fibrosis in MS. Thus, this section (**Figure 5**) begins with an overview of medications that treat fibrosis in non-CNS disorders, and addresses whether these could be applied for MS. We then discuss whether disease modifying therapies (DMTs) used in MS have unintended effects on ameliorating fibrosis in MS, and end with a forward-looking view of potential therapeutics that could be applied to counter fibrosis in MS.

Only two medications are approved to treat fibrosis-related disorders, specifically for lung fibrosis: pirfenidone and nintendanib (5). Pirfenidone acts as an immunomodulator and antagonist of the TGFβ pathway that promotes fibrosis (4, 63). In people with idiopathic pulmonary fibrosis pirfenidone reduced disease progression, improved lung function, and increased exercise tolerance (64). Pirfenidone has undergone phase I and II clinical trials in secondary progressive MS reporting reduced incidence of relapse and improved bladder function (65, 66). However, these trials were small, and a larger study is needed to determine the potential of pirfenidone for people with progressive MS.

When inhibiting TGFβ signaling, it is important to consider its role as a regulatory cytokine and its function for the maintenance of microglia homeostasis in the CNS (44). Another consideration for drugs to overcome CNS fibrosis is the additional challenge that these compounds will have to cross the blood-brain barrier.

The other approved drug for lung fibrosis, nintendanib, is a tyrosine kinase inhibitor (4, 67). Pharmacological inhibition of tyrosine kinases by other drugs in animal models of MS reduced clinical scores, demyelination, inflammation, astrocyte reactivity, and vascular injury (68–71). However, tyrosine kinases are broadly expressed making off-target effects of systemic administration difficult to isolate from potential anti-fibrotic effects within the CNS. Nonetheless, two clinical trials using the tyrosine kinase inhibitor masitinib in progressive MS have been conducted (72, 73). The first, a Phase I trial, showed masitinib to be safe (72). The second, a Phase IIb/III trial, reported reduced elevation of EDSS disability scores and delayed the time to EDSS of 7.0 (73). The extent to which the promising clinical result could be attributed to amelioration of CNS fibrosis is unknown.

Next, we consider whether the approximately (country-dependent) 20 DMTs used in MS have potential impact on evolution of fibrosis (**Figure 5**). While such data is lacking, the immunomodulatory nature (74, 75) of MS DMTs conceivably can alter the immune stimulation of fibrogenesis. Either by reducing the content of pro-inflammatory cytokines or elevating levels of T_h_2 cells (e.g. IFNβ, glatiramer acetate), sequestering leukocytes in secondary lymphoid tissues (e.g. sphingosine-1-phosphate receptor modulators such as siponimod), inhibiting leukocyte trafficking across the blood-brain barrier (natalizumab), or by depleting B cells (anti-CD20 monoclonal antibodies) and the larger leukocyte populations (alemtuzumab, cladribine), the effect would be reduced recruitment and activation of effector cells of CNS fibrosis. Thus, MS DMTs may have unintended and indirect effects on reducing neurofibrosis, although the possibility of this occurring will have to be established. Future work may consider whether the extent of neurofibrosis is ameliorated in people with MS treated with DMTs, and such studies will be highly reliant on evolution of biomarkers with the capacity to detect neurofibrosis.

Recently, inhibitors targeting the intracellular signaling enzyme, Bruton’s tyrosine kinase (BTK), have grown in interest in the treatment of MS (76). Inhibition of BTK in models of graft versus host disease resulted in reduced dermal fibrosis. As well, the first generation BTK inhibitor, ibrutinib, has been approved for treatment of graft versus host disease (77). BTK inhibitors affect B cells but also microglia proliferation, activation, and survival (76). BTK inhibitors have not been trialed for fibrotic disorders, and the preclinical literature is heterogeneous. Some evidence suggests that loss of BTK can reduce fibrosis following cardiac injury while other studies show that BTK inhibitors either have no effect or detrimental effects in kidney and lung fibrosis (78, 79). However, BTK inhibitors have been approved for treatment of several cancers such as chronic lymphocyte leukemia and mantle-cell lymphoma (77). As well, they have been tested in a number of autoimmune diseases leading to many clinical trials including in MS. Five BTK inhibitors have entered late-stage clinical trials including evobrutinib, fenebrutinib, tolebrutinib, remibrutinib and orelabrutanib (76). Unfortunately, evobrutinib failed to meet its primary endpoint in the phase 3 EVOLUTION trials (80). At this time it is unknown whether BTK inhibitors have a role for treatment of fibrosis in MS.

Finally, another means to reduce CNS fibrosis is to prevent or limit the deposition of ECM molecules that occurs after injury. Pharmacological manipulation of ECM and its production have improved recovery in both SCI and MS models (41, 81). Both impairing CSPG synthesis with fluorosamines (4-F- or 4,4-difluoro-N-acetylglucosamine) and digestion of existing CSPGs with chondroitinase-ABC led to significant recovery (41, 81). In EAE, 4,4,-difluoro-N-acetylglucosamine reduced the frequency of cytotoxic T_h_17 and improved remyelination (41). Altering expression of other common ECM components such as hyaluronan improved EAE outcomes (82).

In summary, targeting fibrosis-related responses and outcomes in preclinical studies shows promise as therapeutic strategies for MS. Translation from preclinical into clinically relevant therapies requires more work to understand the implications of these mechanisms on MS and to determine safety and efficacy.

## 7 Conclusions

Tissue regeneration is necessary to restore function (2). Altered immune and tissue remodeling results in fibrosis blocking functional recovery, and increases morbidity (5). The adult CNS does not regenerate well leading to accumulation of injury and disability (16, 83). Inability to repair successfully is partly due to remnant cells and ECM components of fibrosis within the injured CNS (9, 18, 41, 84). Similarities exist between CNS pathologies such as MS and peripheral fibrosis and the benefits of targeting these fibrosis-related processes in MS have been highlighted by a growing body of preclinical research (9, 21, 41, 68) as well as some early clinical trials (65, 66). Fibroblasts are beginning to be recognized as components of neurofibrosis and deserve more studies. Questions remain regarding safety and efficacy of targeting neurofibrosis, but its successful treatment would represent an important step forward in the promotion of CNS regeneration and recovery.

## Author contributions

BL: Conceptualization, Writing – original draft, Writing – review & editing. SG: Writing – original draft, Writing – review & editing. VY: Conceptualization, Funding acquisition, Project administration, Resources, Supervision, Writing – original draft, Writing – review & editing.
